# Triboelectric Nanogenerators for the Masses: A Low-Cost Do-It-Yourself Pulsed Ion Source for Sample-Limited Applications

**DOI:** 10.1021/jasms.4c00010

**Published:** 2024-04-16

**Authors:** Carter K. Asef, Daniel D. Vallejo, Facundo M. Fernández

**Affiliations:** † School of Chemistry and Biochemistry, Georgia Institute of Technology, Atlanta, Georgia 30332, United States; ‡ Petit Institute of Bioengineering and Bioscience, Georgia Institute of Technology, Atlanta, Georgia 30332, United States

## Abstract

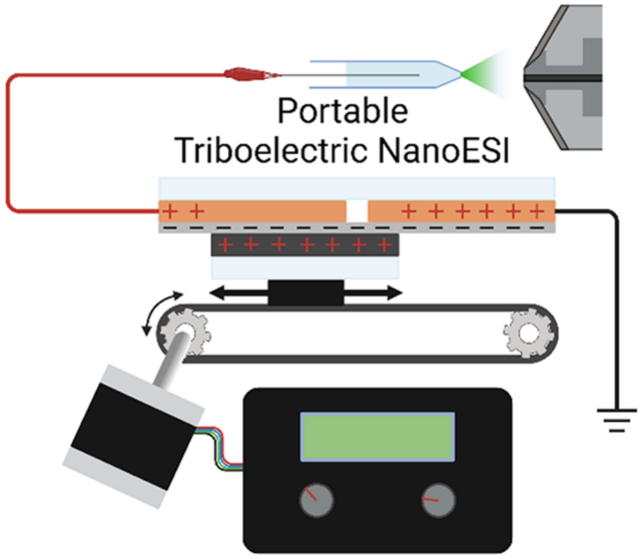

Triboelectric nanogenerators (TENG) are useful devices for converting mechanical motion into electric current using readily available materials. Though the applications for these devices span across many fields, TENG can be leveraged for mass spectrometry (MS) as inexpensive and effective power supplies for pulsed nanoelectrospray ionization (nESI). The inherently discontinuous spray provided by TENG is particularly useful in scenarios where high sample economy is imperative, as in the case of ultraprecious samples. Previous work has shown the utility of TENG MS as a highly sensitive technique capable of yielding quality spectra from only a few microliters of sample at low micromolar concentrations. As the field of miniaturized, fieldable mass spectrometers grows, it remains critical to develop advanced ion sources with similarly small power requirements and footprints. Here, we present a redesigned TENG ion source with a sub-1000 USD material cost, lower power consumption, reduced footprint, and improved capabilities. We validate the performance of this new device for a diverse set of applications, including lipid double bond localization and native protein analysis.

## Introduction

The fundamental operational principle of mass spectrometry (MS) involves the successful generation of gas-phase ions from the analytes of interest. Numerous ionization technologies have been developed that allow probing a diverse range of molecular classes in a variety of ways. Electrospray ionization (ESI) is one of the most widely used methods for investigating biological samples (e.g., metabolites, proteins, etc.), with refinements such as microelectrospray and nanoelectrospray (nESI) offering reduced sample consumption, improved sensitivity, and greatly enhanced ionization efficiency.^1,2^ The majority of ESI approaches, however, rely on high-voltage DC power supplies that apply a constant voltage to the emitter, producing a continuous supply of ions. As a result, ions produced outside of the accumulation or pulsing periods of trapping or time-of-flight mass spectrometers may be wasted and not fully utilized.^3^ Pulsed ESI approaches overcome this drawback, reducing sample consumption with minimal duty cycle losses by rapidly switching current on and off.^4^ In addition, these pulsed techniques reduce emitter heating, allowing for higher voltages to be applied while decreasing the chances of clogging.^5^

Triboelectric nanogenerators (TENG) are intrinsically pulsed voltage generators^6^ and have been previously deployed to improve upon DC nESI approaches.^7−9^ TENG provide voltage to the nESI emitter by harvesting triboelectricity, the electrical potential generated when rubbing dissimilar materials against one another.^10^ This harvested energy can generate very high voltages, on the order of several kilovolts, while delivering nanocoulombs of charge per pulse.^9^ The high voltage/low charge nature of TENG combined with their pulsed implementation enables certain applications that are entirely unique to TENG nESI, such as improving sensitivity for subnanoliter metabolomics^11^ and structural characterization of femtomole quanities of proteins neccessary for cultural heritage objects.^12^ Ion–molecule reactions can also be conducted within the ionization region without damaging the emitter orifice, enabling lipid double bond epoxidation under standard nESI conditions.^13^

Due to their simplicity, TENG have also shown utility in ambient ionization, such as for driving toothpick-ESI to characterize falsified antimalarial medications^14^ TENG are especially useful in this arena, as they preclude the necessity of a high-voltage DC power supply, one of the hurdles in the development of fieldable MS devices that might be operated by untrained personnel.^15^ TENG ion sources use low-voltage power supplies to drive the necessary mechanical motion, with the generated high-voltage electricity presenting little more danger than static shock. As compared to other liquid ionization techniques which also do not require high-voltage power supplies such as atmospheric-pressure photoionization, vibrating sharp-edge spray ionization, and zero-voltage paperspray, TENG is unique in that it can preserve the high sensitivity and other traits associated with nESI when used with standard glass capillary nESI emitters.^11,16−19^

Despite the analytical benefits of TENG for nESI MS analysis, the construction of previous generation TENG nESI ion sources required costly and bulky equipment and materials, restricting their use in field applications and reducing their accessibility. Here, we describe a new TENG nESI ion source build that leverages consumer electronics, bulk materials, and 3D printing. This new construction provides a 20-fold decrease in cost and 30-fold decrease in size compared to previously reported TENG designs, putting it on par with the power supply equipment required for pulsed DC nESI in terms of footprint while maintaining a lower cost.^3−5^ This signifcantly smaller device opens new opportunities for conducting nESI experiments in frugal academic settings, including mobile field demonstrations when powered by a small portable power station or battery. Additionally, this new portability lends well to the growing field of on-site and point of care mass spectrometry.^15,20−23^ We demonstrate this new design is capable of reproducing previously reported data with high fidelity while unveiling new TENG nESI mechanisms not previously reported. We provide extensive step-by-step build instructions with the goal of democratizing TENG nESI for a wider audience in the MS field.

## Materials and Methods

### Chemicals

Lipid standards were purchased through Avanti Lipids. Optima grade water, acetone, and methanol solvents were purchased from Fisher Scientific. LC MS grade ammonium acetate and NIST 1950 reference human blood plasma were purchased from MilliporeSigma. Protein samples were purchased from Sigma-Aldrich in lyophilized form. Cytochrome C (CytC), bovine serum albumin (BSA), concanavalin A (ConA), and alcohol dehydrogenase (ADH) were used as protein standards, and human serum albumin (HSA) was used as a positive control for collision cross-section (CCS) calibration purposes.

### Triboelectric Nanogenerator Device

**Figure 1:**
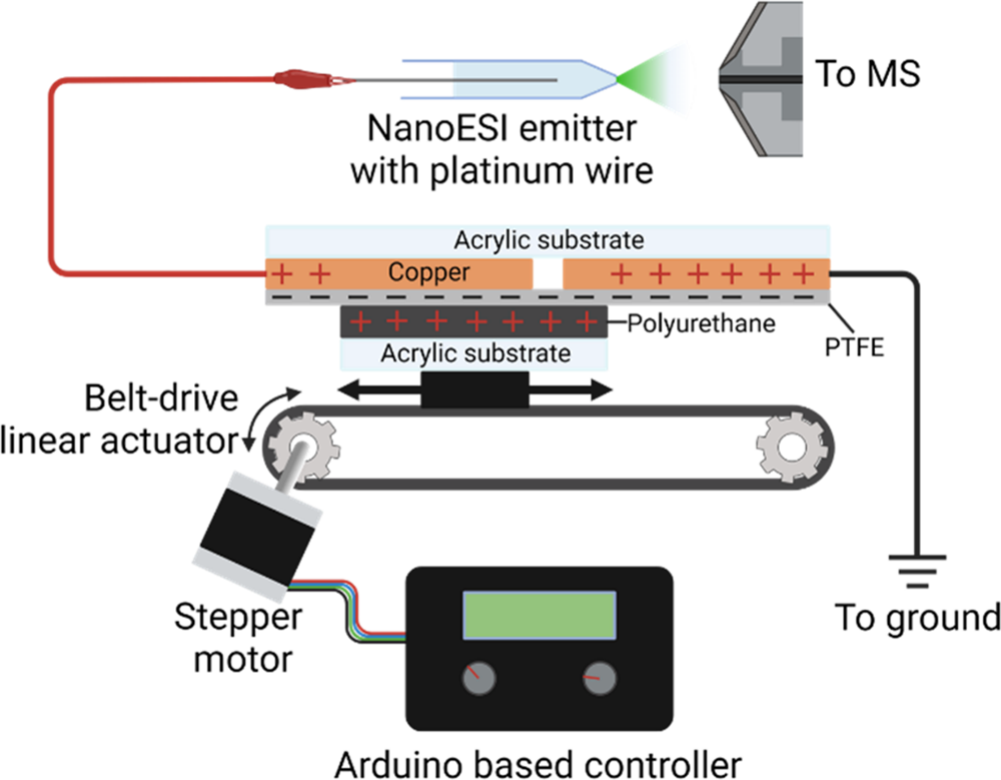
Schematic of the sliding freestanding TENG ion source interfaced with MS. Polyurethane padding is affixed to a rigid acrylic substrate mounted onto the moving platform of a belt drive actuator. The polyurethane film is pressed against a stationary platform of PTFE film and copper tape affixed to a rigid acrylic substrate. The copper tape has a 7.5 mm gap in the middle to form two distinct electrodes, one connected to an earth grounding pin on the mass spectrometer and the other connected to the nanoelectrospray emitter. The belt is driven by an Arduino-controlled stepper motor that slides the moving platform from one end of the stationary platform to the other. The moving platform pauses at each end and then reverses direction for the next stroke. The speed of the stroke and the delay between strokes is controlled by two potentiometer dials connected to the Arduino control board.

The sliding freestanding triboelectric nanogenerator (SF TENG) power source is composed of a stationary platform and a sliding platform (Figure 1). The sliding platform is constructed with a 120 mm × 97.5 mm rectangle of adhesive-backed 1/16′′ polyurethane foam (McMaster-Carr, 86375K162) mounted onto 1/4′′ cast acrylic (McMaster-Carr 8560K354). The stationary platform is a 120 mm × 200 mm rectangle of 1/4′′ cast acrylic covered on one side with adhesive copper foil tape (Amazon, ASIN B095SC3QR7). A 10 mm gap is cut into the copper foil to create two separate 95 mm × 120 mm electrodes at opposite ends of the platform. Small tabs of excess copper foil are left on each of the electrodes to allow for connection to wire leads. A film of 0.01′′ adhesive backed PTFE (McMaster-Carr, 2208T62) is used to cover the entire platform on top of the copper foil. Once both the adhesive and sliding elements are constructed, the sliding platform is mounted onto the moving platform of a belt drive linear actuator (Amazon, ASIN B081Z7S295) using 1/8′′ double-sided foam tape. A cage of 2020 aluminum extrusions is constructed around the actuator to hold the PTFE side of the stationary platform against the polyurethane side of the sliding platform, with uniform pressure. Double sided foam tape is used to mount the stationary platform to the aluminum cage. The linear actuator is controlled by an Arduino Uno R3 housed in a 3D-printed enclosure containing other necessary electrical components. A full list of components and a wiring diagram are shown in Table S1 and Figure S1, respectively. Arduino code, 3D files, and detailed assembly videos can be found on our GitHub (https://github.com/facundof2016/DIY-TENG). The device was powered either by a 24 V 5 A wall power supply or by a 156 Wh, 24 V lithium battery.

### Nanoelectrospray Ionization (nESI) Conditions

Emitters for nESI experiments were produced in-house from borosilicate thin-wall glass capillaries with a starting outer diameter (OD) of 1 mm and an inner diameter (ID) of 0.78 mm (Harvard Apparatus, Holliston, MA). Borosilicate capillary emitters were pulled to ∼10 μm ID for lipid experiments and ∼1–2 μm ID for protein experiments using a Sutter Instruments P-97 Flaming Brown Puller (Novato, CA). Parameters for the puller programs are provided in Table S2. Samples were loaded into the nESI emitter (2–8 μL) using a gel loader pipet, and the emitters were mounted on an x, y, z manual linear stage (Thorlabs, Newton, NJ) to control their position relative to the inlet sampling cone. The emitter tip was held between 7–10 mm away from the inlet orifice. Ions were generated in positive ion mode in all cases, although negative ion mode is also possible.

### Electrical Measurements and Spray Visualization

A Tektronix TDS3012B 1 MΩ oscilloscope was used to obtain waveform data for the TENG device. Due to the low charge generated by the TENG device, a 50 MΩ multimeter was used to better measure maximum voltages. A video (Supporting Information) of the electrospray plume was recorded on a Dino-Lite AM4012PTL microscope camera with illumination from a class 3B 432 nm laser.

### Sample Preparation and MS Analysis

To extract the NIST 1950 standard, 50 μL of plasma was mixed with 150 μL of IPA, vortexed for 1 min, and centrifuged, and the supernatant was transferred to a LC MS vial. Plasma samples were analyzed by LC MS on an Accucore C30 150 × 2.1 mm, 2.6 μm column as previously described.^24^ Collected fractions of reference plasma were dried in a Labconco CentriVap until completely dry and reconstituted at 1/20th the initial volume in 75:12.5:12.5 acetone:water:methanol (v:v:v) with 25 mM ammonium acetate. Lipid standards were first diluted in Optima-grade 2-propanol to concentrations ranging from 200 to 1000 μM and then diluted to their final concentrations of 20 μM in 75:12.5:12.5 acetone:water:methanol (v:v:v) with 25 mM ammonium acetate. Lipid samples were analyzed on a ThermoFisher Orbitrap ID-X tribrid mass spectrometer. Protein samples were analyzed on a Waters Synapt G2 mass spectrometer. All ID-X mass spectra were collected using the ion trap detector in normal scan mode unless otherwise noted. For both mass spectrometers, the standard ion sources were removed and interlocks temporarily defeated as shown in Figures S2–S4. Emitters were held to the inlet of the mass spectrometer using a 3D-printed holder and ring stand (see files in GitHub). Electricity was delivered to the emitters either directly through a platinum wire in contact with the liquid sample within the emitter (Figure 1) or inductively using a conductive graphite sleeve surrounding the emitter (Figure S3). Proteins samples were kept on ice and buffer exchanged into 200 mM ammonium acetate buffer at pH 7 using Micro Biospin columns with either a 6 or 30 kDa cutoff (Bio-Rad, Hercules, CA), based on the analyte protein molecular weight. Buffer-exchanged samples were then diluted to a working concentration of 10 μM prior to analysis.

### Data Analysis

Experimental CCS values for the standard proteins were compared to the standard protein CCS Database.^25^ Experimental CCS values for HSA were calibrated using the standard protein measurements. Percent differences were calculated between the CCS database values, and the average CCS experimental values were computed from intra- and interday CCS measurements. Root-square deviation values were computed for the intra- and interday CCS measurements. Drift time and CCS data were extracted at each collision voltage in DriftScope (Waters, Milford, MA) using TWIMExtract,^26^ and CCS calibrations were conducted using the IMSCal software.^27^

## Results and Discussion

Our previously reported TENG ion source^8^ relied on a linear motor (LinMot, Lake Geneva, WI) to drive the required back-and-forth motion of the sliding polyurethane platform. This design was not suitable for portable or fieldable applications due to the size and power consumption of the linear motor itself and the required ancillary equipment, which included a 72 V power supply, motor controller, and a desktop PC. The first step in reducing the size and increasing portability of this device was to choose a more suitable linear actuator. Belt drive actuators are inexpensive and available in a large number of configurations, making them excellent candidates. The necessary speeds and forces were easily met by a widely available NEMA 23 stepper motor powering the belt drive actuator. From here, the next design choice involved controlling the motor and packaging all ion source elements together. Control functions were accomplished with an Arduino UNO R3 and a mix of readily available electronic, structural, and 3D printed components (Table S1). A schematic of the new TENG ion source is given in Figure 1. Stroke speed and the delay between strokes were controlled using two potentiometer knobs, with the current settings being displayed on the small LCD screen.

**Figure 2:**
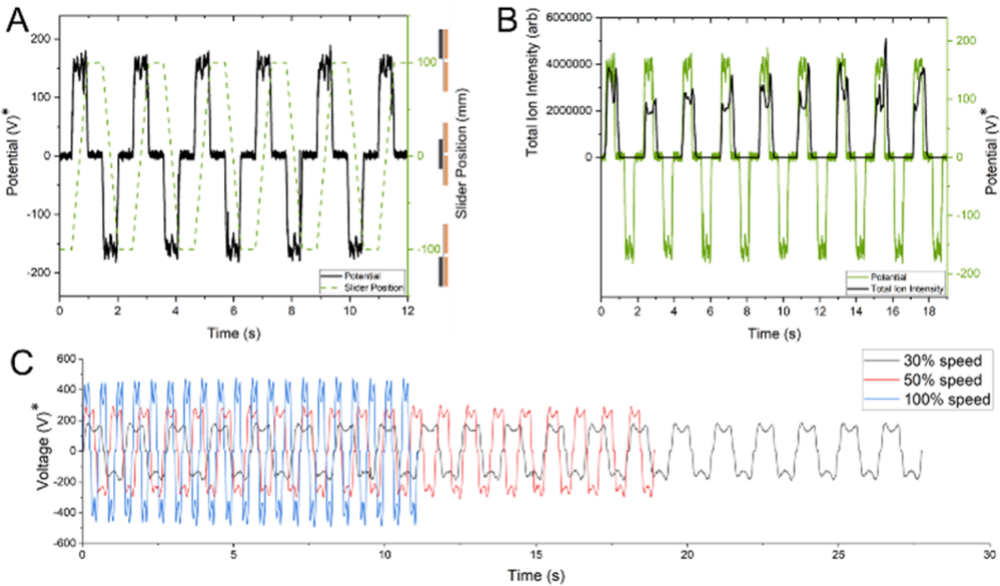
(A) Observed voltage vs slider position demonstrating voltage generation during ion source actuation. The polarity is determined by the direction of motion. (B) An overlay of total ion current vs observed voltage showing that ion signal continues for ∼180 ms after reversal of the electrode motion. (C) A comparison of observed voltages at different stroke speeds over 20 pulses. A stroke speed of 100% represents a frequency of 1.87 Hz. *Voltage as measured by 1 MΩ oscilloscope. Voltages >4 kV measured using a 50 MΩ multimeter. Emitter voltage is likely considerably higher.^9^

Following its construction as documented on GitHub (https://github.com/facundof2016/DIY-TENG), the electrical performance of this new ion source was characterized, given the radically changed construction. Peak voltages were measured at 4.19 kV on the new ion source compared to 3.12 kV on the old source. Due to the low charge generated, it is likely that open circuit voltages are considerably higher than what can be measured on a 50 MΩ multimeter or 1 MΩ oscilloscope.^9^ The reduced voltage of the “older” TENG device may be attributed to aging of the triboelectric materials caused by years of friction, highlighting the need for occasional refreshing of the electrically active layers. A 156 Wh, 24 V battery was able to power the new ion source continuously for ∼8 h at 100% stroke speed, with no delay between strokes. This run time is expected to be considerably longer when operating at reduced stroke speeds with a delay between strokes, as is typical for TENG operation.^28^Figure 2A shows that voltage is generated during the movement of the polyurethane platform, returning to baseline during pauses between motion and then reversing polarity when traveling in the opposite direction. When overlaying total ion counts (TIC) with measured voltage, as in Figure 2B, it was observed that electrospray continued for 3 scans (∼180 ms) after voltage generation had stopped, indicating some degree of accumulated charge in the system was still flowing. Lastly, Figure 2C demonstrates the ability to vary the maximum voltage by changing the sliding speed of the polyurethane platform. The reproducibility of this voltage generation and the correlation to stroke speed are shown in Table 1.

**Table 1:** Maximum Observed Voltage as Recorded for 20 Pulses at Three Speed Settings^a^

	30% speed	50% speed	100% speed
mean potential (V)^b^	182	281	481
standard deviation (V)^b^	3.67	7.93	11.0
RSD (%)	2.0	2.8	2.3
percent max. potential	37.8	58.4	100.0

a The mean, standard deviation, and RSD for these 20 pulses are reported above. The mean potential for 30% and 50% speeds are reported as a percentage of the mean potential at 100% speed in the “percent max potential” fields. A stroke speed of 100% represents a frequency of 1.87 Hz.

b Voltage as measured by 1 MΩ oscilloscope. Voltages >4 kV measured using 50 MΩ multimeter. Emitter voltage is likely considerably higher.^9^

To demonstrate the utility of this new ion source build for bioanalytical purposes, we revisited the previously observed phenomenon of lipid double bond epoxidation during TENG nESI.^8,13^ This epoxidation is caused by operating the TENG ion source in a dual atmospheric pressure chemical ionization (APCI)/nESI mode. APCI is triggered by a pulsed corona discharge that can be observed at the tip of a nESI emitter during negative TENG pulses, when using a platinum wire in direct contact with the liquid solution filling the emitter. When the corona discharge is produced in each cycle of TENG actuation, +16 Da oxidation shifts can be readily observed in the mass spectrum, indicating addition of atomic oxygen. Interestingly, the corona discharge was not observed when using a conductive carbon sleeve surrounding the emitter for inductive charging, indicating the glass emitter causes an effective electric field reduction preventing the breach of the air gap between the emitter and the inlet. Additionally, the low current and pulsed nature of this corona discharge avoids excessive heating of the emitter, therefore not damaging its orifice.

**Figure 3:**
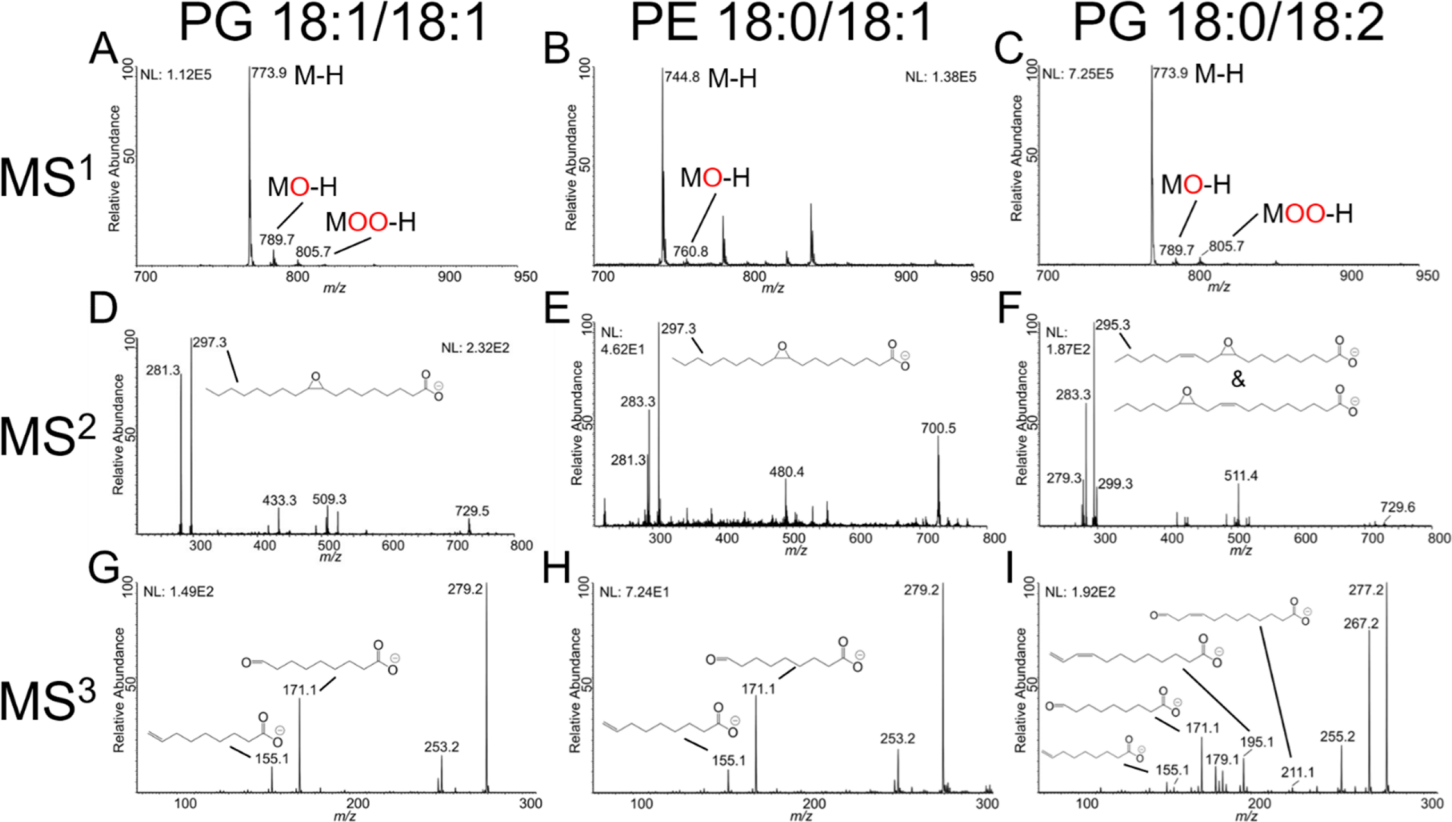
(A–C) Full MS data for TENG nESI analysis of three different lipid standards. The primary observed species are labeled, including singly- and doubly oxidized ions. (D–F) MS^2^ spectra for the singly oxidized lipid species, indicating epoxide formation on unsaturated fatty acid chains. (G–I) MS^3^ spectra for the fragmentation of epoxide chain MS^2^ fragments. Annotated diagnostic fragments indicate the presence of double bonds at the Δ9 position on 18:1 chains, and Δ9 and Δ12 positions of the 18:2 chains.

Oxidation reactions are commonly employed to provide fragmentation sites on unsaturated fatty acid chains, as these lipid C=C bonds do not regularly fragment during collision-induced dissociation. OzID is one such commonly cited technique, though it requires substantial modification to the inner components of the mass spectrometer and the use of hazardous ozone gas.^29^ Other techniques require derivatization, such as Paterno–Büchi reactions,^30−32^ unnecessarily complicating the workflow. TENG nESI is unique in that it requires no additional reagents or modifications to the mass spectrometer other than disconnecting and bypassing the manufacturer’s original ion source and replacing it with the TENG setup. Oxidation reactions are enabled by a specialized sample solvent composition, typically 75:12.5:12.5 acetone:water:methanol with 25 mM ammonium acetate, and direct electrical contact with the liquid sample. The epoxidation reaction can therefore be halted by using different solvents or by using a conductive graphite sleeve to inductively generate the electric field in lieu of the platinum wire. Three lipid standards were purchased with differing lipid class and chain composition as shown in Figure 3. Plots A–C show MS^1^ spectra for each individual standard as measured in negative ion mode using the ion trap detector. Though [M – H]^−^ ions were the primary observed species, a singly oxidized [M + O – H]^−^ feature was observed for all three standards at +16 Da from the primary ion. In both PG standards, which contained a total of two double bonds in their fatty acid chains, doubly oxidized ions were observed as well. Figure 3D–F shows the resulting MS^2^ spectra when isolating the singly oxidized species for each standard. The most intense fragments were the individual fatty acid chains, showing a mixture of oxidized and unoxidized fatty acids. For PE 18:0/18:1, the ratio of the unoxidized 18:1 fatty acid fragment at 281.3 Da to the oxidized fragment at 297.3 Da indicates that a majority of the singly oxidized precursor ion (760.8 Da) was oxidized along the fatty acid chain. These oxidized fatty acid chains were isolated for another round of fragmentation as shown in Figure 3G–I. Fragmentation occurred readily at the site of oxidation to generate aldehyde and aldehyde and terminal alkene fragment ions diagnostic of the double bond position. All annotated MS^3^ fragments aligned with the expected double bond positions for all the purchased standards.

**Figure 4:**
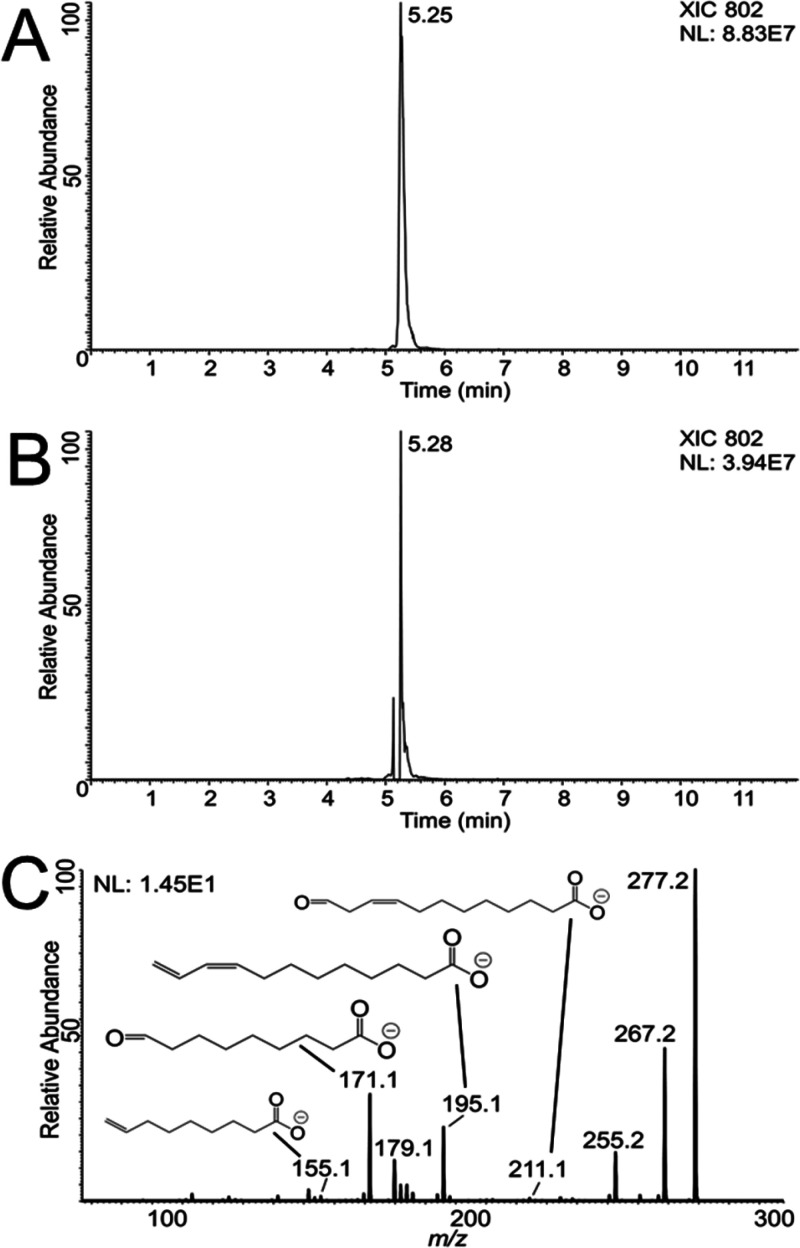
(A) Extracted ion chromatogram (XIC) at *m*/*z* = 802 for (PC 34:2) detected from the LC MS analysis of NIST SRM 1950 reference plasma. (B) A switching valve was employed during elution to divert the feature to a collection vial. (B) XIC of the same feature from a diverted run indicates successful capture of a majority of the feature. (C) MS^3^ spectrum from the collected fraction after drying and reconstituting into 75:12.5:12.5 acetone:water:methanol (v:v:v) with 25 mM ammonium acetate showing diagnostic fragments for double bonds at the Δ9 and Δ12 positions.

Though standards are useful to validate new technologies, their analysis is rarely the end goal of any study. To test the ion source more thoroughly, we used NIST reference serum as a testbed to demonstrate a more biologically relevant application. A single serum sample was extracted with IPA for lipid metabolites and then analyzed by LC MS. Once the retention time was measured for a notable lipid species, PC(34:2), we employed one of the six-way valves of the LC system to divert flow to a collection vial during the elution time of this species. Figure 4A,B shows the XIC for this lipid species before and after employing the divert valve, indicating that the center of the peak was successfully cutoff. Ten injections of 2 μL were diverted for a total collection volume of 400 μL (0.1 min diversions @ 400 μL/min flow rate), which were then dried under vacuum and reconstituted at 1/20th the volume in the oxidation buffer solvent. This concentrated sample was then loaded into a glass capillary emitter and analyzed by TENG-nESI MS^3^. The observed fragments shown in Figure 4C were consistent for PC 16:0/18:2 with the double bonds of the 18:2 chain, identifying it as linoleic acid, a common biological fatty acid. The complete structural elucidation of this lipid from a biological sample shows the ability of TENG-nESI to integrate with traditional LC MS lipidomics studies to provide more detailed structural information on unknown lipids.

The sensitivity of these MS^3^ fragments is the current limiting factor of the technique, as it involves two rounds of fragmentation of a minor oxidation product. Though many parameters of the TENG-nESI experiment including solvent composition, emitter orifice diameter, and TENG stroke settings were optimized to promote oxidation, oxidation rates rarely exceeded 8% of the primary ion. As the number of double bonds in the molecule increases, the ion population becomes further split into more fragment ion channels. These effects ultimately limit the current version of this technology to fatty acid chains with fewer than four double bonds and concentrations in the low-micromolar range or higher.

**Figure 5:**
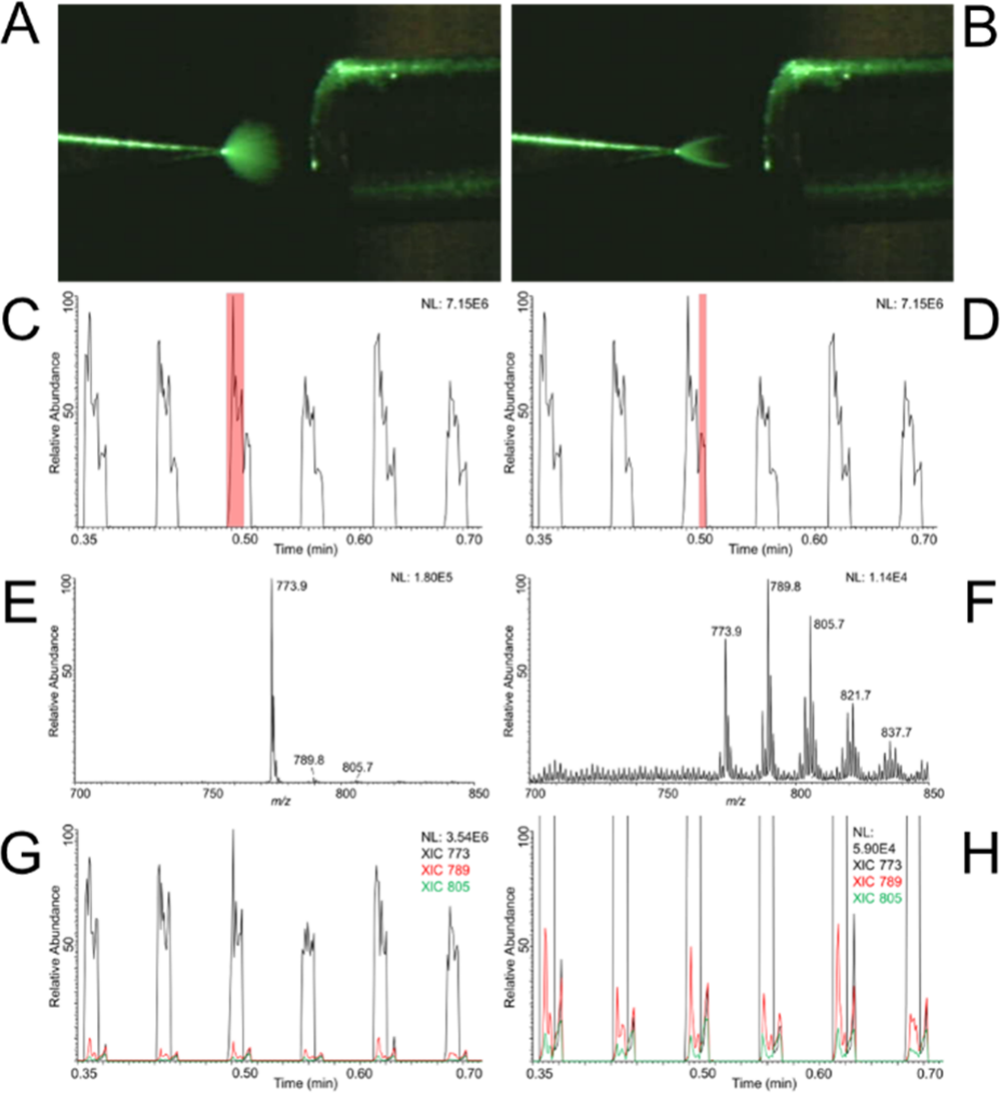
(A) TENG driven spray dynamics earlier in the TENG cycle. (B) A distinct shift in spray mode is observed during the tail end of the voltage pulses when running at low stroke speeds. Regions of normal spray (C) and multijet spray (D) are highlighted with their associated mass spectra (E and F) when analyzing a pure PG(18:1/18:1) standard. Extracted ion chromatograms for [M – H], [MO – H]^−^, and [MOO – H]^−^ are shown (G) and rescaled to oxidized species (H). Extreme ratios of oxidized/unoxidized ions are observed during the multijet regime, though absolute abundances did not increase significantly.

A higher oxidation rate has the potential to increase sensitivity by 1 order of magnitude, greatly expanding the breadth of lipids which could be analyzed by this technique. The oxidation mechanism has been hypothesized to involve hydroxide radicals within the atmospheric pressure corona discharge, though a full investigation has not been performed.^8^ During our analysis, we observed a period of high oxidation yields which occurred during the collapse of the Taylor cone at the end of each TENG pulse. To further investigate this phenomenon, we used the rapid scan rate of the ion trap detector while slowing down the speed of the TENG stroke to 20% with a 1 s delay between strokes (∼0.27 Hz). A recording of the resulting spray is shown in Video S1. Under these conditions, we were able to reproducibly observe [M + O – H]^−^ oxidation products with >100% the abundance of the [M – H]^−^ precursor lipid. The results of this experiment are shown in Figure 5. Though the overall oxidation yield was greatly increased (Figure 5F), the absolute abundance of the oxidized species did not significantly increase (Figure 5H). However, we hope to leverage this phenomenon in the future to better understand the effects of cone formation and collapse on the mechanism of oxidation, improving sensitivity.

**Figure 6:**
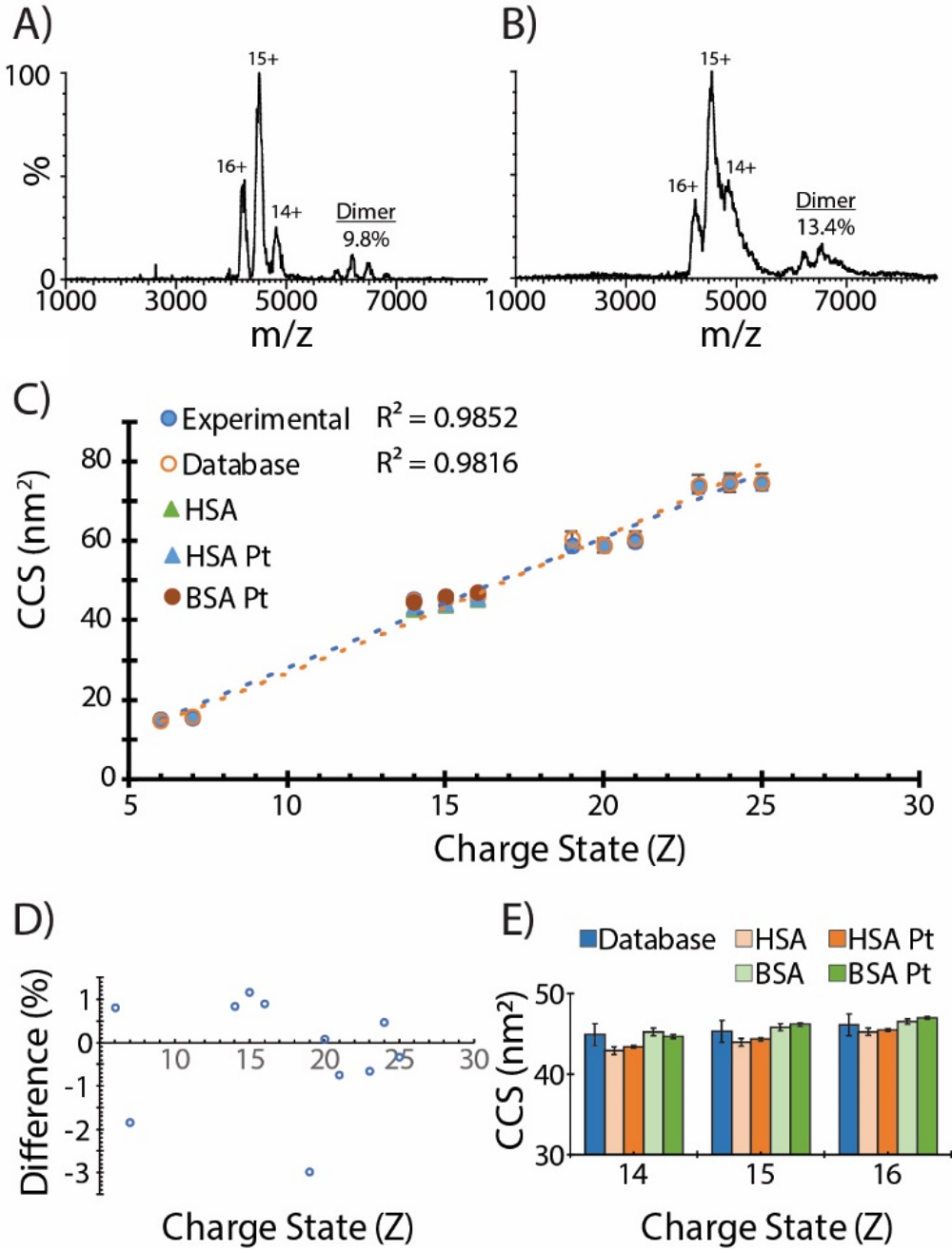
Mass spectra of native BSA under (A) inductive and (B) Pt wire-induced nESI resulting in qualitative differences in peak width (fwhm) and quantitative differences in nonspecific dimers. (C) CCS calibration indicates the new TENG ion source produces high fidelity native protein signals within (D) 3% of database reference values. (E) No quantitative difference in CCS of inductive vs Pt wire-generated protein ions, indicating that in both conditions proteins retain their native structure.

In addition to lipids, we also investigated the performance of this new TENG ion source for protein mass spectrometry. Previously, we reported on TENG-IM-MS approaches and their capability to generate native protein structures with collision cross-sections (CCS) measurements in agreement with community published values. The radically new design of the new TENG ion source warranted re-evaluating its capability to generate high fidelity native MS (nMS) measurements. Using BSA, a well validated protein standard for nMS experiments, the new TENG design resulted in excellent quality native BSA mass spectra (Figure 6A) that were qualitatively and quantitively indistinguishable from the previous TENG data (Figure S5). However, as noted previously, this approach used inductive emitter charging, which did not result in a corona discharge. When using the platinum wire, a mass spectrum that matched the charge state distribution for native BSA was generated but with a notable broadening of the peaks and a slight increase (∼2–3%) in the amount of dimer. These results suggest that the corona discharge is either leading to increased nonspecific oligomerization or to specific oligomerization due to slight protein unfolding. However, when calibrated for CCS we noted no major changes in CCS for BSA or HSA when comparing the conductive or platinum wire ionization approach (Figure 6C–E).

## Conclusions

We present a cost-effective ion source which operates on the triboelectric principle. Detailed instructions for assembly are included in the Supporting Information. This revised design of the TENG meets or exceeds the electrical characteristics of the previous design while reducing cost and footprint. Voltages upward of 4 kV could be measured, and the between-voltage pulse RSD was less than 3% across a range of parameters.

The unique capabilities and simplicity of this ion source make it well suited for its use in a wide range of academic and research environments. The device can interface with most mass spectrometers with atmospheric pressure inlets, ranging from research-grade tribrid Orbitrap systems to simpler quadrupole and ion trap instruments. Its low power consumption (less than 100W) makes it well suited for use with fieldable instruments.

Taken together, we present a cost-effective, field deployable ion source capable of structural characterization for biomolecules ranging from lipids to proteins. By democratizing this technology through build tutorials, we envision other research groups will not only further demonstrate its applications to a wide range of analytes but also innovate upon the technology with unique configurations of their own design.

## Supporting Information Available

Video S1 visualizes nanoESI during the TENG pulses described in Figure 5 (MP4)
Detailed information is provided on the TENG hardware list, wiring diagram for assembly, MS inlet setup, and data comparison between old and new TENG designs (PDF)
