# Supplementary material for: Triboelectric Nanogenerators for the Masses: A Low-Cost Do-It-Yourself Pulsed Ion Source for Sample-Limited Applications
Source: J Am Soc Mass Spectrom. 2024 Apr 16;35(5):943–50. doi: 10.1021/jasms.4c00010 (PMC11066968; doi:10.1021/jasms.4c00010)
Supplement: Supplementary file 2 — js4c00010_si_002.pdf [file js4c00010_si_002.pdf]

## Supporting Information for:

# Triboelectric Nanogenerators for the Masses: A Low-Cost Do-It-Yourself Pulsed Ion Source for Sample-Limited Applications.

Carter K. Asef[1], Daniel D. Vallejo[1], Facundo M. Fernández[1,2]*

[1]School of Chemistry and Biochemistry, Georgia Institute of Technology, Atlanta, Georgia 30332, USA

[2]Petit Institute of Bioengineering and Biotechnology, Georgia Institute of Technology, Atlanta, Georgia 30332, USA

*Email: Facundo.Fernandez@chemistry.gatech.edu

# Table of Contents

**Table S1.** List of hardware components ........................................................................................ **S3**

**Table S2.** Program settings for P-97 pipette puller........................................................................ **S5**

**Figure S1.** Wiring diagram for device electronics ........................................................................ **S6**

**Figure S2.** Source setup for use with Thermo Orbitrap ID-X ...................................................... **S7**

**Figure S3** Source setup for use with Waters Synapt G2-S ........................................................... **S8**

**Figure S4.** Alternate source setup.................................................................................................. **S9**

**Figure S5.** Comparison of new device with old device for native protein analysis.................................. **S10**

**Table S1**. List of components for TENG ion source assembly. Most parts will accept generic replacements, though part numbers are provided for specific components where relevant. Total cost of listed components was USD 662.53 as of January 2024.

| Part name | Notes | Vendor | Part ID | Price (USD) |
|---|---|---|---|---|
| Elegoo UNO R3 | any Arduino UNO clone | Amazon | ASIN B0B6VV7MS7 | 16.99 |
| Heechoo motorized high speed belt drive linear guide rail (200 mm) | any NEMA 23 driven 200 mm belt drive. Must be >65 mm travel per rotation | Amazon | ASIN B081Z7S295 | 159 |
| TB6600 motor controller | any motor controller with direction pin and pulse pin. Needs to tolerate 36V and 3 A | | | 9.98 |
| 24V 4A power supply | must match power jack size, any >24 V >3 A power supply will work | | | 24.99 |
| 5.5x2.5 mm female power jack | must tolerate at least 100 W | | | 9.99 |
| Optical limit switch | EE-SX674, Heechoo brand will fit the Heechoo belt drive without additional components | Amazon | ASIN B082FFNH5P | 36 |
| 20 90-degree brackets for 2020 extruded aluminum | | Amazon | ASIN B07GDVV2SR | 21.97 |
| 150 mm T-slot extruded aluminum x16pcs | 200 mm extrusions are also acceptable | | | 47.96 |

| | | | | |
|---|---|---|---|---|
| male female pin crimps | | Amazon | ASIN B0146DJR9Q | 7.88 |
| on/off boat rocker switch | | Amazon | ASIN B07Y1GDRQG | 5.99 |
| 4.8 mm female Baomain connectors x2pcs | | | | 6.99 |
| b10k 10 k ohm linear taper potentiometer x2 pcs | | | | 9.99 |
| LM2596 buck converter | must convert 24 V to 7-9 V | | | 5.49 |
| colored wire assortment 22 AWG | 22 AWG | | | 14.99 |
| 1/4" cast acrylic 12"x12" | | McMaster-Carr | part 8560K354 | 18.38 |
| 0.01" adhesive backed PTFE, 12"x12" | | McMaster-Carr | part 2208T62 | 24.32 |
| 1/16" polyurethane foam sheet with adhesive back "medium" hardness 12"x12" | | McMaster-Carr | part 86375K162 | 11.18 |
| 1/8" thick double sided foam tape, 1/2" wide | 1/4" thick tape is preferable if available | | | 18.99 |
| 6" copper foil tape | | Amazon | ASIN B095SC3QR7 | 16.99 |
| 1602 lcd display | 1602A | | | 8.99 |

| | | | | |
|---|---|---|---|---|
| ring stand with burette clamp | | | | 19.99 |
| M5 x 10 mm screws x4pcs | | | | 9.99 |
| Four M2.5 x 4 mm screws x4pcs | | | | 8.79 |
| ESI Conductive Sleeve | | Waters | SKU 700000969 | 17 |
| Platinum wire | | ThermoFisher | catalog number 045093.BW | 92.2 |
| Premium Thin Wall Borosilicate with Filament | | Warner Instruments | model G100TF-4 | 37.5 |
| 3D printed Arduino case | https://www.printables.com/model/438969-arduino-uno-project-box-case | | | |
| 3D printed emitter holder | https://www.printables.com/model/529755-nano-electrospray-ioniziation-nanoesi-emitter-hold | | | |

**Table S2**. Sutter Instruments P-97 Flaming Brown Puller (Novato, CA) program settings for achieving desired orifice sizes.

| Program | Ramp Temp | Heat | Pull | Velocity | Delay | Cycles |
|---|---|---|---|---|---|---|
| 10 µm lipid | - | 570 | 0 | 20 | 1 | NA |
| 1-2 µm protein | 524 | - | NA | 26 | 1 | 1 |

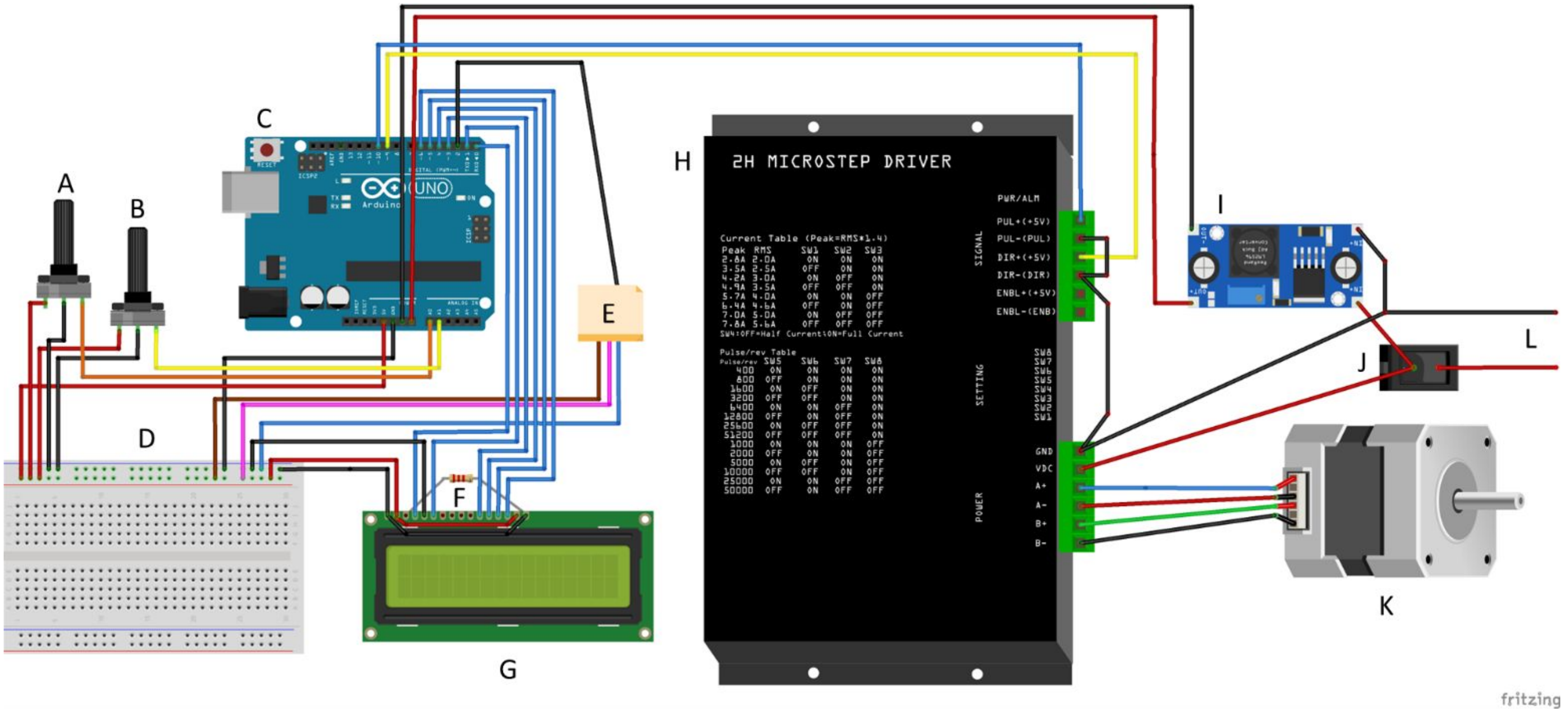


**Figure S1.** Wiring diagram for the assembly of TENG ion source electronics. (A) potentiometer to control speed, (B) potentiometer to control stroke delay, (C) Arduino UNO R3 powered by VIN and GND input, (D) breadboard shown to demonstrate wiring of 5 V and ground rails. Join all ground wires and 5 V leads together with wire nuts in lieu of breadboard. (E) EE-SX674 Optical limit switch, (F) 2 kΩ resistor to set contrast, (G) 1602 LCD screen, (H) TB6600 stepper motor driver set to 1600 Pulse/rev and 2.8 A, (I) LM2596 buck converter with output voltage set to 7 V, (J) Power switch, (K) Nema 23 stepper motor, (L) to 24 V power supply capable of >3 A.

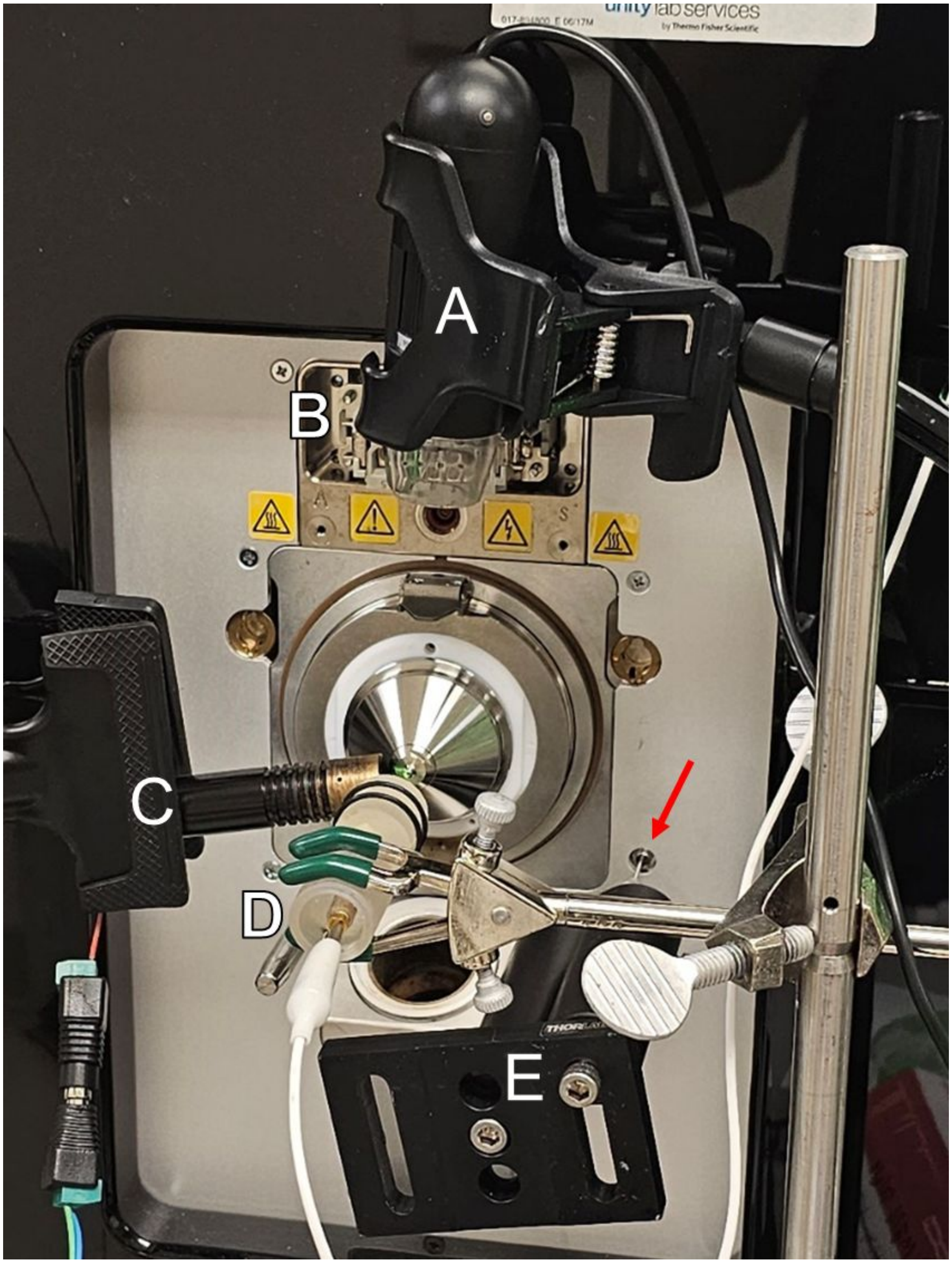


**Figure S2.** Detail of the setup in the ion source region for TENG nESI analysis in a Thermo ID-X mass spectrometer. (A) Microscope camera for recording spray dynamics (optional). (B) Allied electronics part #09200102612 with a 10 kΩ resistor across pins 10 and 6 and jumper wire across pins 4 and 9 (caution, exposed high voltage). (C) Laser to help visualize spray for microscopy camera (optional). (D) 3D printed emitter holder with platinum wire insert. (E) Various Thor Labs post holders and brackets used

to hold down the button highlighted with a red arrow. One electrode of the TENG device is wired to the emitter (D), while the other electrode is wired to the frame of the TENG device and to the grounding pin of the MS power supply. Most LC MS systems have numerous grounding pins on the rear of the instrument.

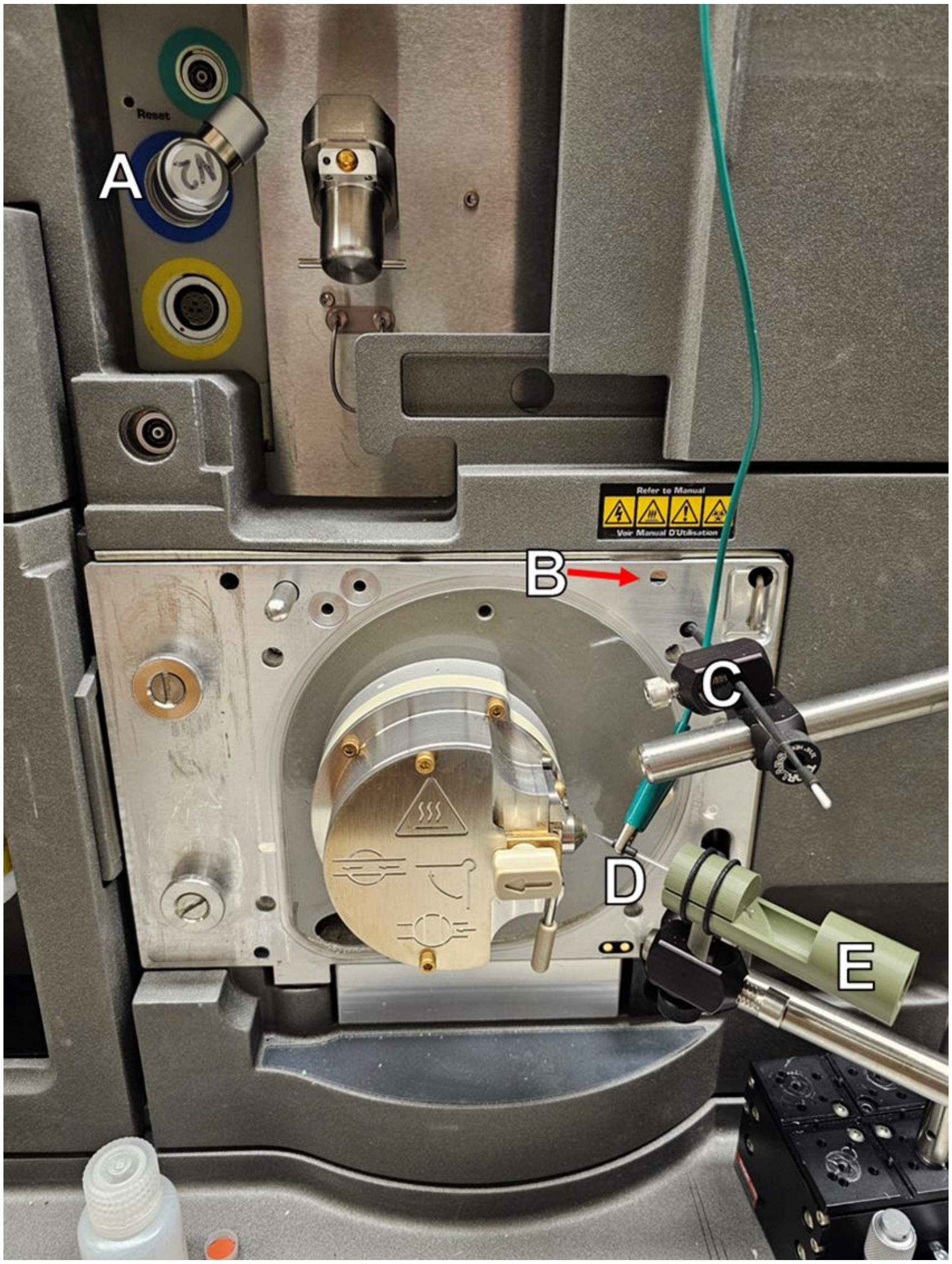


**Figure S3.** Detail of the setup in the ion source region for TENG nESI analysis on a Waters Synapt G2-S. (A) Kensington Electronics part number WS0 105 A038-130+ with a 1 kΩ resistor across pins 16 and 17 and a jumper wire across pins 16 and 18. (B) Source door sensor, which must be pressed in by a mix of ring stand parts (C). (D) Glass nanospray emitter with a

conductive graphite sleeve to inductively apply voltage to the emitter. (E) 3D printed emitter holder cartridge, held in place by ring stand or Thor Labs XYZ stage.

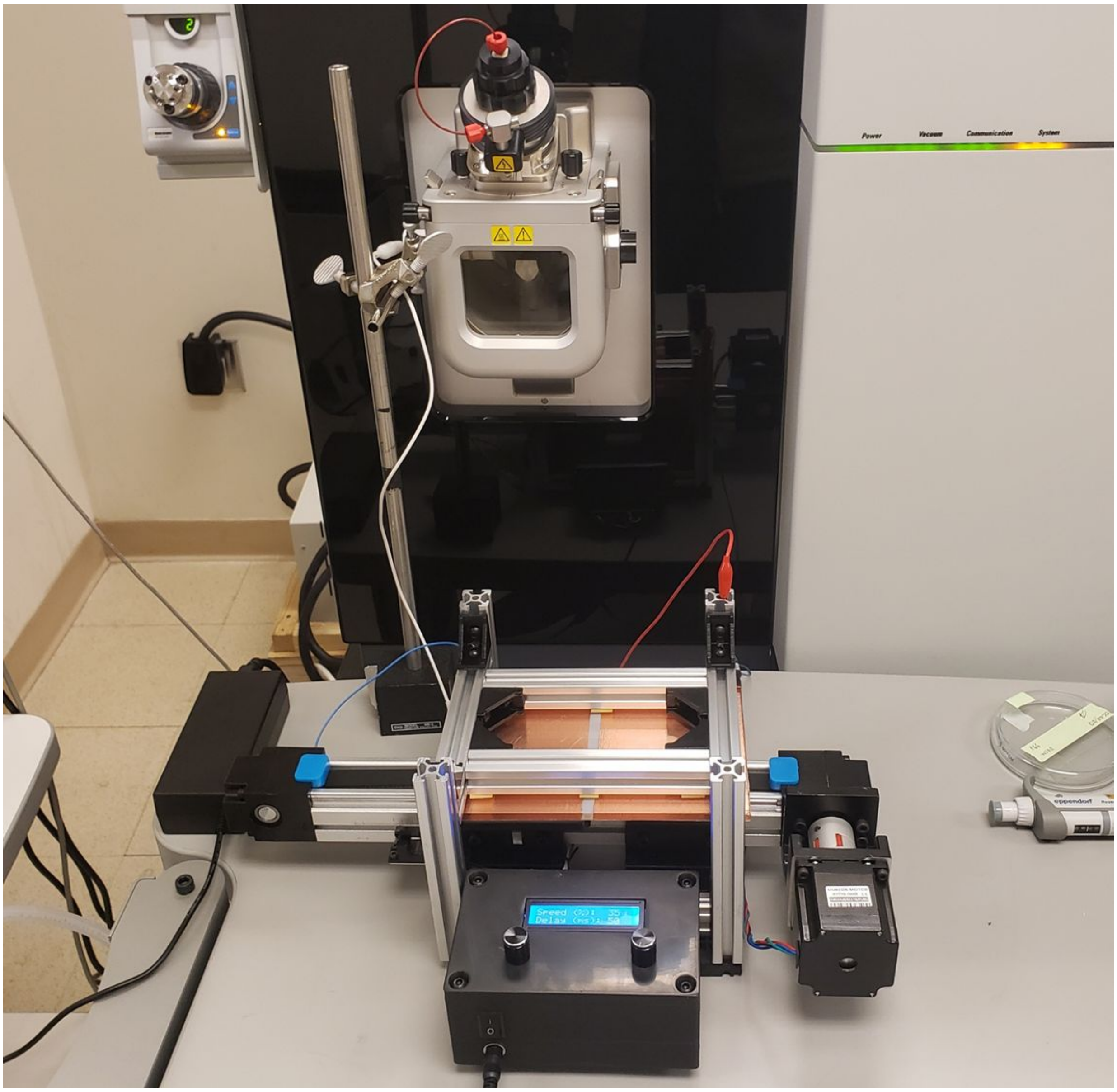


**Figure S4.** Alternate TENG setup keeping the ion source housing in place. Spray voltage is set to 0 V within MS software, and the emitter and holder are inserted into the side port of the housing.

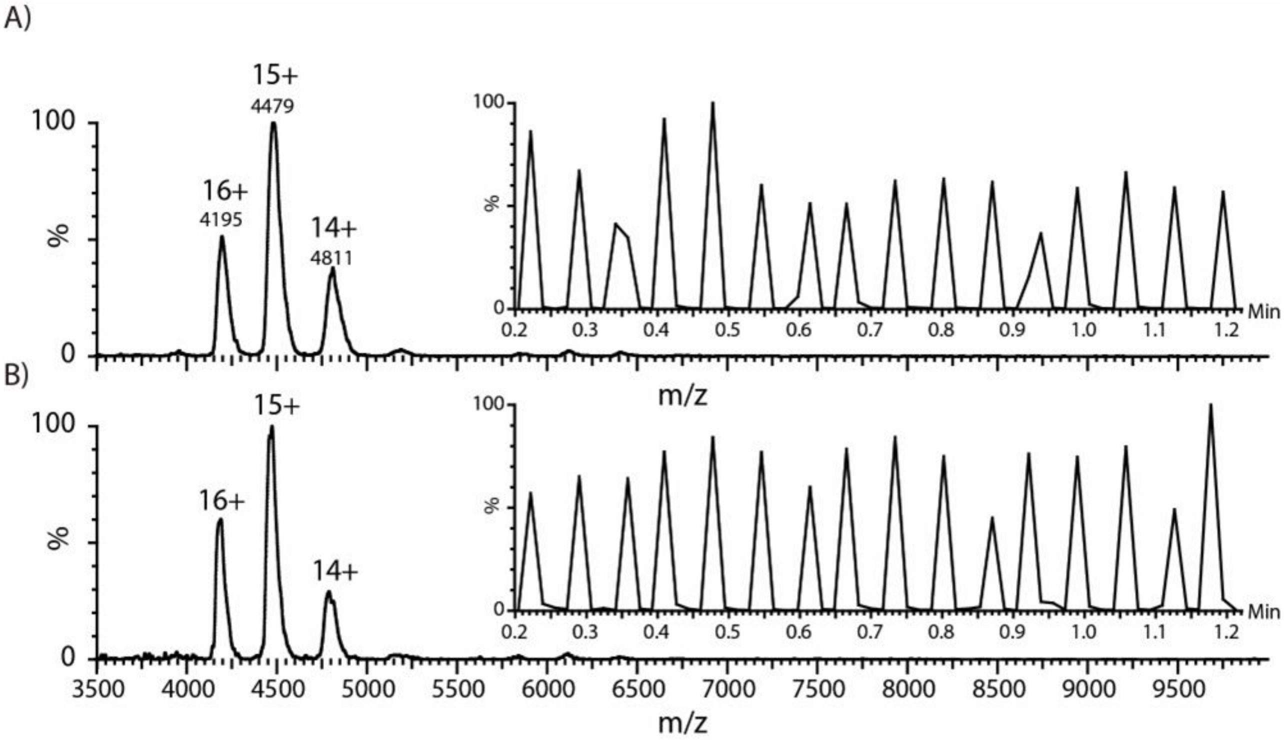


**Figure S5.** Pulse-to-pulse variability in ion intensity and native mass spectral quality for the new TENG ion source construction (A) is comparable to those for the (B) original TENG design, without any discernable losses in the analytical capabilities.
